# Human Health Risks Related to Penicillin G and Oxytetracycline Residues Intake Through Beef Consumption and Consumer Knowledge About Drug Residues in Maroua, Far North of Cameroon

**DOI:** 10.3389/fvets.2020.00478

**Published:** 2020-08-26

**Authors:** Ronald R. B. Vougat Ngom, Harquin S. Foyet, Rebecca Garabed, André P. Zoli

**Affiliations:** ^1^National Advanced School of Engineering, University of Maroua, Maroua, Cameroon; ^2^Zabit Laboratory of Agronomy and Geography, Maroua, Cameroon; ^3^Department of Biological Sciences, Faculty of Sciences, University of Maroua, Maroua, Cameroon; ^4^Department of Veterinary Preventive Medicine, College of Veterinary Medicine, The Ohio State University, Columbus, OH, United States; ^5^School of Veterinary Medicine and Sciences, University of Ngaoundéré, Ngaoundéré, Cameroon

**Keywords:** dietary intake, penicillin G, oxytetracycline, Cameroon, risk assessment

## Abstract

In Cameroon, a leading livestock production country, previous study highlighted the extensive misuse of veterinary drugs in the Far North Region, where we found the second cattle population of the country. Based on former work describing the presence of penicillin G (PEN) and oxytetracycline (OTC) residues in beef sold for human consumption in Maroua, the capital city of the Far North Region, this study was designed to determine the risk of PEN and OTC residue intake via beef consumption by the population of Maroua and to evaluate the consumer's knowledge on drug residues. Dietary exposure determined here was based on the average consumption of beef (found during a survey of 202 households randomly selected in Maroua) and the average concentration of the two antibiotic residues in beef (obtained after liquid chromatography–tandem mass spectrometry analysis of samples of liver and muscle collected from 202 cattle selected randomly in all the slaughterhouses of Maroua) found in our previous studies. The estimated daily intakes per capita of OTC and PEN residues from beef consumption were 22.81 and 2.37 μg, respectively. The risk was then assessed as high [9.6%; range from 6.4 to 15.4% of acceptable daily intake (ADI)] and considerable (2.2% range from 1.5 to 3.5% of ADI) for PEN and OTC residues, respectively. Based on the mean value, it can be concluded that the estimated risk of antibiotic daily intake through beef in Maroua is high (5.9% of ADI). Also, 98% of the surveyed population is not aware that meat can be contaminated by residues of veterinary drugs administered to slaughter animals. This ignorance would also increase the risk of contamination of the population of Maroua with antibiotic residues. This work clearly highlights the high risk of consuming beef by the population of Maroua. Taking into consideration the evidence of the intake of antibiotic residues from others sources, it is therefore very imperative that measures be taken by the government to ensure food safety in order to guarantee the health of the populations of this region in particular and Cameroon in general, hence the need for the establishment of a national food safety plan.

## Introduction

Recent statistics showed that Sahelian countries are mainly meat consumers, and it is estimated that demand for animal products in this area is expected to increase by more than 250% by 2025 (1). In Cameroon, where 36% of country's 24 million people live below the poverty line and 17.1 million persons were food-insecure in 2018 (2, 3), food of animal origin constitutes an important part of the population's diet (4). Indeed, in Cameroon, livestock is an important sector of the national economy. In the Far North Region, the second most populated region in the country, livestock farming is a traditional activity for several ethnic groups (5). In Maroua, the biggest city of that region, beef is an important part of the population's diet. Indeed, meat is consumed in 96% of households, and 98% of them consume beef. The daily consumption of beef per capita in this area is estimated at 133 g, which is 10 times higher than the worldwide average meat consumption (6, 7).

However, a recent study conducted in this region showed that the meat of more than 20.30% cattle slaughtered is contaminated with the residues of two antibiotics named penicillin G (PEN) and oxytetracycline (OTC). These are the mostly used veterinary drug in this region (8, 9). The average concentration of these antibiotic residues in beef was estimated at 17.58 μg/kg for PEN and 240 μg/kg for OTC (9). This is in accordance with Food and Agriculture Organization/World Health Organization reporting that antimicrobial residues edible animal products have grown beyond permissible level or are very high in developing countries (10). These residues may be harmful to human health (disruptions of normal intestinal flora, allergic reactions, mutagenic effect, risk of teratogenicity, carcinogenicity, etc.) after intake of contaminated beef (11). In fact, foods of animal origin containing residues of drugs are considered a public health risk (12). It is also well-known that most of the antimicrobial use in livestock can lead to development of antimicrobial-resistant bacteria in muscle food, which can then be transmitted to humans via food and other transmission routes (13, 14).

Studies conducted in several countries have highlighted the level of risk faced by populations consuming food of animal origin containing antibiotic residues (15–17). For example, in Lebanon, the risks due to tetracycline and penicillin residue intake from all dairy products were estimated at 0.007 and 0.006%, respectively (18). Bou-Mitri et al. (19) from their study in the same country found that the dietary exposure to penicillin through meat consumption was equivalent to 88.3, 31.9, and 5.7% of the acceptable daily intake (ADI), using Tiers 1, 2, and 3 approaches, respectively. In Iran, Aalipour et al. (20) found the risk of dietary exposure to tetracycline residues via bovine milk intake varied from 7 to 30% of the determined ADI. Unfortunately, at the level of our knowledge, to date, no such studies have been carried out in Cameroon. Yet foodborne diseases and other food safety issues are among the greatest development challenges we face globally. In Africa, it is estimated that 92 million people fall ill from consuming contaminated foods, resulting in 137,000 deaths each year (21).

In order to ensure consumer safety, food safety authorities of many countries and organizations all over the world have tried to prevent or reduce the negative health effects of veterinary drugs residues on consumers by establishing regulations. The Codex Alimentarius Commission has recommended an ADI of 30 μg of PEN/capita per day and 0–30 μg of OTC/kg body weight (BW) per day (22). In Cameroon, because of the importance of this issue, the first law on food safety was passed on December 11, 2018, but no specific laws exist on food of animal origin. In fact, food safety is a particularly important issue and is one of the priorities of the national nutrition policy in Cameroon. Indeed, improving food safety is a requirement that stems partly from Cameroon's ambition to strengthen its trade position at the international level, but also to consolidate its position at the subregional level. In addition, there is an internal demand for improved food safety from a public opinion mobilized on this theme (23).

Taking the fact that in the Far North of Cameroon beef is one of the mostly consumed meat and that beef contain antibiotic residues, it is therefore indicated that in this region the risk level of antibiotic residue intake through beef consumption can be higher. The aim of this study was to determine the risk of OTC and PEN residue intake via beef consumption by the population of Maroua in the Far North Region of Cameroon and to evaluate the consumer's knowledge on drug residues. Dietary exposure assessment is a critical step in assessing the public health risk posed by these residues.

Risk assessment is generally carried out in four stages (24): hazard identification, hazard characterization (including dose–response assessment), exposure assessment (human exposure), and risk characterization (description of the nature and extent of the risk to humans). The first step of this assessment in the Far North Region of Cameroon was made during our previous work (8). The second step was also addressed during the studies conducted in the same area (6, 9). This paper is then the last two steps of this risk assessment.

## Materials and Methods

### Study Area

This research was conducted in 2014 in the urban area of Maroua, the capital city of the Far North region of Cameroon. Maroua is the chief town of the Diamare Division with a surface area of 4,665 km^2^. The population of Maroua was 403,817 in 2013 (Report of the Far North Regional Delegation of Economy, Planning, and Regional Development, 2013, not published). Geographically, the city of Maroua is located between latitude 10–13° North of the Equator and longitude 13–15° East of the Greenwich meridian (25).

### Data on Beef Consumption

The food consumption data used in this study were based on the existing and representative data of our previous study (6). During that study, 202 households selected using a gripped map and random selection method as described by Vougat Ngom et al. (25) were surveyed. The number of households investigated was calculated and set, taking into consideration the economic feasibility of the study but also considering the results of the presurvey done in the city of Maroua. The cookers or cooks were surveyed and observed in each household. During this survey, the pieces of beef, as cuts before cooking, were weighted, and the quantity of beef consumed per capita was calculated. The influence of socioeconomic and demographic factors related to consumer (religion, age, monthly income, number of persons in the household, and district) on beef consumption was also evaluated. The results of the quantity of beef consumed per capita in Maroua found in that study can be seen in the Data Sheet 1 (Supplementary Material).

During the survey, the weight of all the members of each household selected was recorded by using a scale to determine the variation of BW among population according to their socioeconomic and demographic parameters. Because of low literacy rates in the study area, informed consent was verbally obtained before proceeding with each survey. The guardian(s) of children enrolled in the study also provided verbal, informed consent for the weighing of their children, and at least one guardian was present during the weighting. Each home and individual household member was assigned unique identification codes to ensure anonymity. In a household, participants were made aware that they were free to stop the survey at any time, for any reason and without penalty. The permission to perform this work in households was granted from the local Cameroon Ministry of territorial administration and traditional community leaders.

### Data on Penicillin G and Oxytetracycline Residues in Beef

The data of the concentration of PEN and OTC residues in beef used in this study were based on our previous study (9). During that study, to determine the mean concentration of antibiotic residues in beef, samples of liver and muscle were collected from 202 cattle selected randomly in all the slaughterhouses of Maroua and Godola and analyzed using liquid chromatography–tandem mass spectrometry according to the revised method of the US Department of Agriculture (26) followed by some modifications. Characteristics of the cattle selected (age, sex, breed, body condition score, weight, production system, pathology, etc.) were also collected before and/or after slaughter by physical examination and survey and postmortem examination. The average residues concentration in beef calculated during that study was 17.58 μg/kg for PEN and 240 μg/kg for OTC. The results of the concentration of antibiotic residues on beef according to cattle demographics found during that study can be seen in Data Sheet 1 (Supplementary Material).

### Estimated Daily Intake

Dietary exposures from consumption of beef contaminated with antibiotic residue were estimated, and the estimated daily intake (EDI) was calculated by the following equation described by Aalipour et al. (20). Dietary exposure here is based on the average consumption of beef and the average concentration of contaminant in this food (27). To do all the calculations, the results of our previous study on beef contamination with drug residues were used (9).

$$\begin{array}{r} \text{EDI}=\text{Beef consumption }\left(\text{g}/\text{day}\right)\;\times \text{ average concentration of residues in beef} \end{array}$$

### Level of Achievement of Accepted Daily Intake (Risk Scoring)

The values obtained from the previous equation, which reveal the level of exposure of the urban population of Maroua to OTC and PEN residues in beef, were then compared to the ADI values set by Codex Alimentarius Commission at a concentration of 0–30 μg/kg BW per day for OTC and 30 μg/person per day for PEN in cattle meat products using the following equation (20):

$$\begin{array}{l} \%\text{ ADI}=\text{100 }\times \text{ intake}(\text{μg/day})\;÷[(\text{ADI})(\text{μg/kg BW per day})\\ \;\times \text{ BW}(\text{kg})] \end{array}$$

Based on the estimated intake and comparison with the ADI, the risk was assessed as negligible when the “level of achievement” was <1% of ADI (<1% of ADI), as considerable if “level of achievement” was 1–5% of ADI (1–5% of ADI), and as distinctively if “level of achievement” was >5% of ADI (>5% of ADI) (28).

### Estimating Acute Exposure: Global Estimated Acute Dietary Exposure

Global estimated acute dietary exposure (GEADE) was calculated based on the 97.5th percentile food consumption amount using the equation as (16):

$$\begin{array}{r} \text{GEADE}=\frac{97.5\text{th percentile food consumption }\times \text{ high residue tissue}}{\text{BW}} \end{array}$$

The 97.5th percentile food consumption amount was used because of its statistical robustness and is more representative than the maximum food consumption amount (29).

### Evaluation of the Population's Knowledge of Drug Residues

Studies have shown that there is a relationship between the population's dietary knowledge and dietary habits. For this purpose, to assess the knowledge of the population of Maroua of the problem of veterinary drug residues, questions relating to this subject were asked during the household surveys carried out as described by Vougat et al. (6).

### Data Analysis

The average antibiotic residue intake per capita in Maroua according to his socioeconomic and demographic parameters were compared by performing the one-way analysis of variance followed by the multiple-comparisons test of Tukey by using GraphPad Prism version 5.00 for Windows (GraphPad Software, San Diego, CA, USA; www.graphpad.com). Survey data were analyzed by using the Statistical Package for Social Sciences (SPSS) software version 20.0 (IBM® SPSS® Statistics, http://www.ibm.com/support~software).

## Results

### Average Weight of an Individual in Maroua

Table 1 summarizes the weight variation of the population of the study area according to their socioeconomic and demographic characteristics. During the survey, from the 202 households, the weight of population was recorded in 85, in whom the consent was verbally obtained. It appears that the average weight of an individual in the population in Maroua is 49.28 ± 19.40 kg. This weight is not affected by sex and household size. However, a household's monthly income and age significantly affect the average weight (*P* < 0.001).

**Table 1:** Average body weight (in kilograms) of individuals according to their socioeconomic and demographic characteristics.

**Variables**	**Body weight**	**Significance**	**Sample size**
Religion		*	
Christian	50.03 ± 24.62^a^		273
Muslim	42.90 ± 24.09^b^		142
Animist	49.00 ± 30.28^ab^		4
Sex		NS	
Male	46.30 ± 25.84^a^		176
Female	47.13 ± 24.78^a^		257
Age (years)		***	
0–15	24.57 ± 13.84^b^		191
16–45	65.28 ± 15.26^a^		196
>45	65.18 ± 16.30^a^		42
Household size		NS	
<5	49.60 ± 25.93^a^		102
5–10	46.43 ± 24.95^a^		289
>10	42.43 ± 24.84^a^		42
Monthly income ($)		***	
Low (<30)	35.36 ± 22.02^b^		65
Average (30–120)	49.83 ± 24.63^a^		208
High (>120)	50.13 ± 24.58^a^		150
Average weight	49.28 ± 19.40		434

The values represent the averages weight of an individual ± SEM. For the same variable, values with different letters of the alphabet in the same column are significantly different (P < 0.05); NS, not significant difference at the 5% level. *P < 0.05; ***P < 0.001. For variables with two modalities, the comparison was made by performing the Mann–Whitney test, but for those with more than two modalities, the one-way analysis of variance followed by the Tukey multiple-comparisons test was performed.

### Daily Intake of PEN and OTC Residues From Eating Beef

The variation of the average amount of PEN and OCT residue intake per capita in Maroua through beef consumption is presented in Table 2. Results showed that the average daily intake of PEN residues from eating beef per capita was estimated at 2.37 ± 0.23 μg (varies from 1.52 ± 0.43 to 3.39 ± 0.34). This dairy intake is influenced by age and religion (*P* < 0.01). The highest daily intake of PEN was observed in the oldest age group (3.39 ± 0.36 μg) and from people in households with high monthly incomes (2.92 ± 0.19 μg).

**Table 2:** Daily intake (μg) of antibiotic residues per capita in Maroua.

**Variable**	**PEN**	**OTC**	**Significance**	**Significance**
			**PEN**	**OTC**
Religion				
Muslim	2.75 ± 0.10^a^	38.02 ± 2.85^a^	**	**
Christian	2.06 ± 0.12^b^	28.23 ± 3.13^b^		
Age (years)			**	***
0–15	1.52 ± 0.43^a^	20.98 ± 11.82^a^		
16–45	2.68 ± 0.31^b^	35.32 ± 21.54^b^		
>45	3.39 ± 0.34^ab^	44.00 ± 24.25^ab^		
Household size			NS	NS
<5	1.59 ± 0.28^a^	21.63 ± 7.24^a^		
5–10	1.90 ± 0.55^a^	26.19 ± 14.02^a^		
>10	2.04 ± 0.62^a^	21.98 ± 14.34^a^		
Monthly income ($)			NS	NS
Low (<30)	2.79 ± 0.44^a^	38.09 ± 5.97^a^		
Average (30–120)	2.71 ± 0.30^a^	37.01 ± 4.04^a^		
High (>120)	2.92 ± 0.19^a^	39.87 ± 2.61^a^		
**Mean**	**2.37** **±** **0.23**	**32.08** **±** **8.08**		

Each value represents the average daily intake of antibiotics per capita in Maroua ± SEM; NS, no significant difference; the values of the same variable with different letters of the alphabet in the same column are significantly different (P < 0.05), ***P < 0.001; **P < 0.01.

The average daily intake of OTC residues per capita in Maroua ranges from 20.98 to 44.0 μg. The average amount of OCT intake per day is 32.08 ± 8.08 μg. This daily intake rate varies with the age of the person (*P* < 0.001) and according to his religion (*P* < 0.01).

### Risk Assessment to Antibiotic Residues

Table 3 presents the risk variation to which the people of Maroua are exposed after beef consumption according to their socioeconomic and demographic characteristics. Thus, in general, the risk associated with the daily consumption of PEN in Maroua found is 9.6% of ADI. This risk is very high (estimated intake >5% ADI). People who are at highest risk are those belonging to households with low monthly income (risk per day = 15.4% of ADI), people younger than 16 years (risk = 12.4% of ADI), and Muslims (risk per day = 11% of ADI).

**Table 3:** Risk (% of ADI) associated with the antibiotic residue intake in beef.

**Variable**	**Penicillin G**	**Oxytetracycline**
Religion		
Muslim	11	2.5
Christian	9.6	2.2
Age (years)		
0–15	12.4	2.9
16–45	8.2	1.8
>45	10.4	2.3
Household size		
<5	6.4	1.5
5–10	8.2	1.9
≥10	9.6	1.7
Monthly income ($)		
Low (<30)	15.4	3.5
Average (30–120)	9.2	2.1
High (>120)	9.2	2.1
**General mean**	**9.6**	**2.2**

However, the risk associated to the daily intake of OTC residues in beef in Maroua estimated is 2.2% of ADI. This risk is considerable (1% < estimate risk <5% of ADI). The highest and lowest risks were recorded in low monthly income households (3.6% of ADI) and children younger than 5 years (1.5% of ADI), respectively.

### Global Estimated Acute Dietary Exposure Assessments

The GEADE for the antibiotic drugs residues studied has not yet been established. Computed GEADE of each antibiotic residue was therefore compared to its ADI. The GEADE of PEN varied from 0.21 μg/kg BW per day (40% ADI) to 4.5 μg/kg BW per day (900% ADI). The GEADE for OTC varied from 0.05 mg/kg BW per day (160% ADI) to 0.13 mg/kg BW per day (260% ADI). The higher GEADE of PEN and OTC was recorded in Muslim people (Table 4).

**Table 4:** Global estimated acute dietary exposure assessments associated with the antibiotic residue intake in beef.

**Variable**	**PEN (μg/kg BW per day)**		**OTC (mg/kg BW per day)**
	**Liver**	**Muscle**	**Liver**
Religion			
Muslim	4.50	0.52	0.13
Christian	3.00	0.35	0.08
Age (years)			
0–15	3.57	0.42	0.10
16–45	3.32	0.39	0.09
>45	3.42	0.40	0.10
Household size			
<5	1.84	0.21	0.05
5–10	3.58	0.42	0.10
≥10	3.43	0.40	0.10
Monthly income ($)			
Low (<30)	3.62	0.42	0.10
Average (30–120)	2.75	0.32	0.08
High (>120)	2.88	0.34	0.08

*GEADE, Global Estimated Acute Dietary Exposure. OTC was not found in muscle samples*.

### Population Knowledge on Drug Residues in Animal Products

It came from the field work that only 1.53% (three of 196) of the surveyed households are informed that meat can be contaminated with residues of veterinary drugs administered to slaughter animals. The majority (66.67% or two of three) of those who know this have learned it at school. They are also the ones who know the consequences of these residues on the consumer's health.

## Discussion

Results revealed that the average amount of PEN residues daily intake per capita of Maroua during beef consumption is not negligible (2.37 ± 0.23 μg) compared to the maximum residue limit 30 μg (22). Indeed, this intake of antibiotic residue is essentially linked to the consumption of beef. Because several other sources of intake of this antibiotic residues exist in the study area (milk, meat from small ruminants, chickens, egg, etc.), the total amount of residue intake per capita is probably greater than that observed. This high amount of drug residue intake is the obvious consequence of the misuse of veterinary drug by the cattle farmers in Far North of Cameroon (8). In addition, the daily intake of residues of PEN is more than nine times the daily intake of the same drug in southwestern Nigeria (0.005 mg/kg per day) (15). This difference is due to the fact that the quantity of beef consumed in Maroua is higher (134 g) with respect to that registered by these authors (38 g). The estimated dietary intake found in this study is also higher than that observed in Lebanon (9.6 μg/kg BW per day) by Bou-Mitri et al. (19).

From the analysis of data, it came out that, in Maroua, the daily intake of OTC residues per capita via beef consumption is 32.08 ± 8.08 μg (or 0.65 ± 0.16 mg/kg per day). This rate varies according to religion, age, and monthly income of the household. The estimated intake of OTC calculated is higher than the intake of tetracycline residues observed via milk consumption of high consumers in Iran (1.65 μg/kg BW per day) (20).

The daily intake of antibiotic residues recorded in this study can be reduced after cooking this beef. Indeed, several studies including those of Abou-Raya et al. (30) and Heshmati (31) have shown that temperature and cooking method considerably reduce the amount of penicillin residues in food. However, the method used to assess the risk in this study is acceptable because it allows a quick understanding of possible risks to human health as a result of taking food with residues of veterinary medicines.

Nevertheless, the rate of antibiotic residue consumption estimated could affect the health of consumers. Indeed, several studies have shown that intake of PEN residues can cause allergic reactions, hypersensitivity, and anaphylactic reactions in sensitive consumers (32, 33). This is important, given that ~5–10% of the population worldwide is hypersensitive to PEN (34). This can also reduce consumers' confidence and result to adverse impact on country's economy. Finally, one of the major problems that can arise from this consumption is bacterial resistance to antibiotic (11), which is a public health problem and a challenge of our time due to the current and potential impact on global population health, costs to healthcare systems, and gross domestic product, mainly through reduced treatment options (35, 36). Indeed, infections caused by resistant bacteria result in longer duration of illness, higher mortality rates, and increased costs associated with alternative treatment (37). Current projections suggest that antimicrobial resistance will cause ≈300 million premature deaths by the year 2050 (35). A work carried out in our study area had already highlighted the presence of some antibiotic resistance genes in bacteria (38). This has raised an alarm to the Cameroonian government who is already taking steps to address this problem. Indeed, because of the importance of this issue, a framework law on food safety was passed on December 11, 2018. In addition, some still very limited actions carried out in the field by certain government projects and non-governmental organizations (Zabit Laboratory of Agronomy and Geography) contribute to raising awareness among livestock farmers on the proper use of veterinary medicines.

Based on the results, the risk from PEN residue intake through beef was assessed as high (9.6% of ADI). This risk is also high compared to what has been observed in several developing countries such as Nigeria (15) and in developed countries such as Brazil (17) and Croatia (39). This divergence of results is due to the fact that the consumption of beef in Maroua is very high compared to what is observed in those countries.

The risk associated with the daily intake of PEN residues via beef consumption in Maroua is higher in Muslim households and in households with low monthly income. It is also higher for people younger than 16 years. This is due to the high consumption of beef and low BW of people of these different classes.

Contrary to what was observed in the case of PEN, the risk evaluate from OTC daily intake in Maroua is considerable (2.2% of ADI). This probability reinforces the fact that this issue is a real problem in the study area. This risk is greater than that obtained in Nigeria (0.024%) by Adesokan et al. (15). This dissimilitude of results is partly due to the fact that the quantity of beef consumed in Maroua exceeds the Nigerian consumption, but in addition, the average concentration of OTC residues in beef sold in our study area is high compared to what is recorded in this country of West Africa.

In comparison to the study conducted in Ghana (16), the GEADE estimated in this study is sometime 260% of ADI. This implies that the concentrations of the antibiotic residues found in the samples present acute toxicity to consumers of Maroua.

Results showed that 98% (or 193 of 196) of the surveyed population are not aware that meat can be contaminated by residues of veterinary drugs administered to animal, and only 1.53% are aware of the consequences that would result from the consumption of such meat. This result could be explained by the fact that in this part of Northern Cameroon, ~40% of the population is poor (40) and therefore has little access to school, which is the place where one can be informed of this type of problem. Indeed, there is a correlation between the level of education and knowledge of dietary concepts (41). However, according to Van Loo et al. (42), in the United States, what motivates people to buy more organic chicken is that they are sure that it contains fewer residues (hormones, antibiotics, etc.).

## Conclusion

The results obtained in this study showed that the daily intake per capita of PEN and OTC residues in the city of Maroua following the consumption of beef is considerable when compared to the ADI recommended by the Codex Alimentarius. This daily intake varies according to the age of the consumer, religion, and the monthly income of the household to which he/she belongs. The risks associated with this daily intake of residues are 9.6% of ADI and 2.2% of ADI for PEN and OTC, respectively. Based on the mean value, it can be concluded that the estimated risk of antibiotic daily intake through beef in Maroua is high (5.9% of ADI). The socioeconomic and demographic factors mentioned above also increase the risk of exposure to PEN residue intake. Indeed, low income households are the most at risk, followed by children under 16 and Muslims. In addition, 98% of the surveyed population are not aware that residues of veterinary drugs administered to animals can contaminate meat. This lack of knowledge of this issue could increase the probability of veterinary drug residue intake via the animal products, mainly because in this study area there is an evidence of the intake of antibiotic residues from others sources (chicken, meat of small ruminants, milk, eggs, etc.). The risk associated with antibiotic intake estimated in this study can be reduce during cooking of beef as demonstrate by many authors. Further studies on this risk by using a cocked beef and a high sample of household is needed to have a more accurate risk assessment. In despite of that, the results of this study clearly highlights the presence of a real risk of consuming beef by the population of Maroua. This indicates that, consumers of beef in Maroua are predisposed to health hazards and hinders international meat trade from Cameroon. Until today in 2020, no national program has been introduced to monitor the presence of drug residues in animal products in Cameroon. The results of the present study can be informative for the Government to instigate new policies to restrict the present potential risk in order to guarantee the health of the populations of this region in particular and Cameroon in general.

## Data Availability Statement

The data used/or analyzed during the current study are available from the corresponding author on reasonable request.

## Ethics Statement

The University of Maroua approved this study and the permission to perform this work in households was granted from the local Cameroon Ministry of public service and administrative reform and traditional community leaders. Because of low literacy rates in the study area, informed consent was verbally obtained before proceeding with survey. The guardian (s) of children enrolled in the study also provided verbal, informed consent for the weighing of their children's and at least one guardian was present during the weighing.

## Author Contributions

This paper is a part of the Ph.D. dissertation of the first author. It is the product of an interdisciplinary collaboration between the University of Maroua (Cameroon) and the Disease Ecology and Computer Modeling Laboratory of the Ohio State University (Columbus, OH, USA). The study was conceived and designed by RV, HF, and RG. AZ supervised the fieldwork. RV collected, analyzed the data, and wrote the manuscript. All authors read and approved the final manuscript.

## Conflict of Interest

The authors declare that the research was conducted in the absence of any commercial or financial relationships that could be construed as a potential conflict of interest.

## Abbreviations

PEN: Penicillin G
OTC: Oxytetracycline.
