# Supplementary material for: Human Health Risks Related to Penicillin G and Oxytetracycline Residues Intake Through Beef Consumption and Consumer Knowledge About Drug Residues in Maroua, Far North of Cameroon
Source: Front Vet Sci. 2020 Aug 26;7:478. doi: 10.3389/fvets.2020.00478 (PMC7480011; doi:10.3389/fvets.2020.00478)
Supplement: Supplementary file 1 [file Data_Sheet_1.pdf]

Table 1. Socio-economic and demographic characteristics of the interviewees in the household (6).

Variables	Percentage (%)	Sample size (No. of household)
Age (years)$^{+}$		202
< 10	0	
[11-15]	4.1	
[16-25]	26.9	
[26-45]	53.8	
>45	15.2	
Religion^{++}		201
Christian	55.2	
Muslim	43.8	
Animist	1.0	
Marital status		200
Single	12.0	
Married	81.0	
Divorcee	1.5	
Widowed	5.5	
Educational level		200
No formal education	32.0	
Koranic	10.5	
Primary	28.0	
Secondary	24.5	
University	5.0	
Family income		158
< $30	15.2	
[$30-$120]	44.3	
> $120	40.5	
Average number of people in the house	6.37	202

($^{+}$): age of the principal interviewee; (++): religion of the head of the household;
Sample correspond to the number of household and each of them has an average of 7 persons.

Table 2. Some variables of meat consumption (6).

Variable	Percentage (%)	Sample size (No. of households)
*Eat meat***	95.50	202
*Meat more eaten***		199
Beef	72.36	
Sheep	5.02	
Goat	20.60	
Chicken	1.01	
Pork	1.01	
*Eat beef***	97.90	193
*Frequency of beef consumption**		196
Every day	10.7	
Twice or three times a week	38.8	
Once a week	12.8	
Twice or three times a month	10.7	
Once a month	4.1	
Rarely	22.4	
Do not know	0.5	

*$P<0.05$, **$P<0.01$; Sample correspond to household and each of them has an average of 7 persons.

Table 3. Quantity of beef consumed per person in Maroua (6).

Variable	Per day (g)	Per month (g)
Quantity of beef consumed per person	133.25 ± 33.49	1296.50 ± 239.41
Age		
0-15	92.78± 6.22^{c}	524.99 ± 119.80^{b}
16-45	151.84 ± 15.63^{b}	1151.10 ± 86.83^{a}
> 45	179.22 ± 40.93^{a}	1332.63 ± 329.72^{a}
Household monthly income+		
Low	157.21 ± 11.92^{a}	1306.65 ± 129.48^{a}
Average	129.77 ± 19.50^{b}	1477.16 ± 380.86^{a}
High	130.0 ± 12.17^{b}	1429.70 ± 152.05^{a}
Religion		
Christian	135.39 ± 10.09^{a}	1861.00 ± 731.62^{a}
Muslim	136.66 ± 15.24^{a}	857.87 ± 32.10^{b}
Number of person in the household		
<5	94.08 ± 17.76^{a}	684.13 ± 155.40^{a}
5≤n<10	94.78 ± 8.08^{a}	579.42 ± 152.49^{a}
≥10	79.96 ± 44.50^{a}	569.00 ± 265.56^{a}

(+) Correspond to the monthly income of the head in the household; Low, less than $30; Average,$30-$120;High, more than $120. The values represent the average consumption of beef in g. For the same variable, values with different letters of the alphabet in the same column are significantly different ($P<0.05$).

Table 4. Percentage (%) of cattle carcasses contaminated according to their demographics (9).

Variables	PEN	OTC	OTC+PEN
Age (year)			
< 8 (n = 37)	18.92	0	18.92
8-10 (n = 101)	17.82	1.98	19.80
> 10 (n = 59)	16.95	1.69	18.64
ND (n = 5)	60	0	60
Sex			
Entired male (n = 13)	15.38	0	15.38
Castrated male (n = 4)	0	0	0
Female (n = 185)	19.46	1.49	21.08
*Production system**			
Transhumance (n = 16)	25	6.25	31.25
Sedentary (n = 62)	19.35	0	19.35
*Type of production**			
Fattery (n = 46)	17.39	0	17.39
Mixed (n = 30)	23.33	3.33	26.67
ND (n = 125)	18.4	1.60	20
*Health status***			
Sick (n = 21)	80.95	4.76	85.71
Healthy (n = 181)	11.60	1.10	12.70
Body condition score			
Thin (n = 131)	18.32	1.53	19.85
Normal (n = 69)	18.84	1.45	20.30
Fat (n = 1)	100	0	100
*Breed**			
Goudali (n = 13)	23.08	0	23.08
White Fulani (n = 65)	18.46	1.54	20
Red Fulani (n = 96)	20.83	2.08	22.92
Arabe Shuwa (n = 8)	12.5	0	12.5
Toupouri (n = 3)	33.33	0	33.33
Kapsiki (n = 9)	11.11	0	11.11
Peul (n = 1)	0	0	0
ND (n = 7)	0	0	0
Slaughterhouses	13.04	1.74	14.78
Municipal (n = 115)	22.22	0	22.22
Makabaye (n = 45)	26.92	0	26.92
Kongola (n = 26)	25	12.5	37.5
Meskine (n = 8)	0	0	0
Salak (n = 5)	100	0	100
Godola (n = 3)			

PEN : penicillin G ; *OTC* : Oxytetracycline; *ND*: No determined; *P<0.05, **P<0.01

Table 5. Concentration (µg/g) of penicillin residues on beef according to cattle demographics (9).

**Variable**	**Mean**	**Median**	**Maximum**	**Minimum**
Sex				
Male (n = 3)	0.109	0.068	0.198	0.068
Female (n = 35)	0.140	0.072	0.773	0.001
Age (year)				
< 8 (n = 7)	0.108	0.094	0.198	0.009
[8 ; 10] (n = 17)	0.106	0.013	0.773	0.001
> 10 (n = 14)	0.192	0.106	0.734	0.003
Breed				
Red Fulani (n = 21)	0.143	0,072	0,773	0,001
White Fulani (n = 12)	0.144	0.067	0.734	0.001
Goudali (n = 2)	0.061	0.061	0.068	0.054
Kapsiki (n = 1)	0.088	/	/	/
Arabe Shuwa (n = 1)	0.119	/	/	/
Toupouri (n = 1)	0.182	/	/	/
Tissus				
Muscle (n = 5)	0.054	0.055	0.090	0.009
Liver (n = 33)	0.151	0.087	0.773	0.001
Body condition scoring				
Fat (n = 2)	0.128	0.128	0.196	0.061
Normal (n = 13)	0.164	0.087	0.734	0.001
Thin (n = 23)	0.124	0.066	0.773	0.001
Health status				
Sick (n = 16)	0.163	0.090	0.773	0.001
Healthy (n = 22)	0.120	0.062	0.734	0.001
Type of production				
Fattering (n = 8)	0.096	0.061	0.337	0.001
Mixed (n = 6)	0.263	0.211	0.773	0.003
ND (n = 24)	0.121	0.070	0.734	0.001
Production system				
Sedentary (n = 12)	0.144	0.067	0.773	0.001
Transhumance (n = 3)	0.227	0.303	0.325	0.054
ND (n = 23)	0.123	0.072	0.734	0.001

ND: No determined
